# Concurrent overexpression of serum p53 mutation related with *Helicobacter pylori *infection

**DOI:** 10.1186/1756-9966-29-65

**Published:** 2010-06-04

**Authors:** Juan-Bosco Lopez-Saez, Victoria Gómez-Biondi, Germán Santamaría-Rodriguez, Margarita Dominguez-Villar, Antonio Amaya-Vidal, Antonio Lorenzo-Peñuelas, Avelino Senra-Varela

**Affiliations:** 1 Department of Medicine, Puerto Real Medical College, University of Cadiz, c/. Dr. Marañón n°6 11003, Cadiz, Spain

## Abstract

**Background & Aims:**

In the province of Cadiz (Spain), the adjusted mortality rate for gastric cancer in the coastal town of Barbate is 10/100.000 inhabitants, whereas in the inland town of Ubrique, the rate is twice as high. The rate of *Helicobacter pylori *(*H. pylori*) infection (*H. pylori *antibodies) in the normal population was 54% in Ubrique, but only 32% in Barbate. In the two decades since its original discovery, p53 has found a singularly prominent place in our understanding of human gastric cancer and *H. pylori *cause accumulation of reactive oxygen species in the mucosa compartment. This study was designed to compare serum levels of p53 in a population characterized by high mortality due to stomach cancer and a high prevalence of *H. pylori *infection and another population in which mortality from this cause and the prevalence of *H. pylori *infection are low.

**Materials and methods:**

319 subjects from the low mortality population and 308 from the high mortality population were studied, as were 71 patients with stomach cancer. We measured serum immunoglobulin G antibody to *H. pylori *and serum mutant p53 protein and ceruloplasmin.

**Results:**

The difference between the two populations in the prevalence of *H. pylori *infection was significant (p < 0.001). Of the seropositive, 81% had elevated values of mutant p53, in comparison with 11% of the seronegative (p < 0.0001). Serum concentration of ceruloplasmin was significantly higher in seropositive with elevated mutant p53 protein than in seronegative with normal levels of p53 (p < 0.05).

**Conclusions:**

There is a significant association between infection with *H. pylori*, elevated titers of *H. pylori *antibodies, and positivity for serum mutant p53 protein. Such information can significantly increase our basic knowledge in molecular pathology of gastric cancer and protection against *H. pylori *infection.

## Introduction

Mortality due to gastric cancer in Spain has decreased markedly since the period from 1960 to 1965, but remains high in some mountain locations [1]. In the southern Atlantic province of Cadiz, coastal towns such as Barbate have an adjusted mortality rate of 10/100.000 inhabitants, whereas towns such as Ubrique, located in the mountainous region 30 kilometers inland, have an adjusted mortality rate of 20/100.000 [2]. An earlier study found that the rate of Helicobacter pylori infection (determined by measuring serum *H. pylori *IgG antibodies) in the normal population was 54% in Ubrique, but only 32% in Barbate, where the mortality rate for stomach cancer is lower. Mean antibody titers are also higher in the area with the higher mortality rate [2].

*H. pylori*, originally under the genus Campylobacter [3], is a ubiquitous bacterial pathogen that infects more than 50% of the world's population. *H. pylori *was first cultured in vitro, and shown to be associated with gastritis and peptic ulcers, by Marshall and Warren [4]. *H. pylori *infection in untreated subjects is usually lifelong, and the ongoing chronic infection can to be an etiological agent of chronic gastritis, peptic ulcer disease and carcinoma [5]. Chronic infection with *H. pylori *affects approximately half the world and results in malignancy in a small subset of this population. Although the frequency of infection in developed nations is falling with a resultant decline in *H. pylori*-associated peptic ulcer disease, gastric cancer remains the second major cause of cancer death worldwide, with *H. pylori *infection being a major attributable factor in the development of gastric cancer [6]. Research into the relationship between the two is ongoing, however, suggested that between 35 and 55% of all gastric cancers may be related to *H. pylori *infection [7].

Since 1994, the International Agency for Research on Cancer (IARC) designated *H. pylori *as a class I human carcinogen, it now is well accepted that gastric infection by *H. pylori *is a risk factor for development of gastric cancer [8]. Although the precise pathogenetic role of *H. pylori *in gastric carcinogenesis remains unclear, it has been clarified that this organism contributes to modifications in epithelial cell proliferation, which may be the initiating event in a cascade culminating in the development of gastric cancer [9], but it is not known whether the increased risk is due to the presence of mutant p53 generated by chronic gastritis or to a direct action of the bacteria on the p53 oncogene [10,11].

The p53 gene mutation is associated with approximately 70% of tumors of different orignis [12,13]. p53 gene serves as a "gatekeeper of the cell", for its role in preventing the accumulation of genetic alterations through the regulation of critical checkpoints between the end of G1 and the beginning of S to redirect cells with a mutation in the genome toward apoptosis or programmed cell death. This key oncogene thus prevents the perpetuation of a defective genome and the development of a cancer [14].

Several recent studies have been published on the presence of p53 in patients with *H. pylori *infection, stomach cancer, or both. However, the conclusions are contradictory, and it has been difficult to develop a single coherent hypothesis that can account for all findings communicated to date [15]. Palli et al [16] found p53 mutants in 33 of 105 cases of gastric cancer and Domek et al [17] worked with the hypothesis that tumorigenesis involves deregulation of cell proliferation and apoptosis. These researchers investigated cell proliferation and apoptosis in the gastric epithelium of children infected with *H. pylori*, and found that the apoptotic index was significantly higher in patients with *H. pylori *gastritis than in patients with secondary gastritis or healthy control subjects. They also reported that apoptosis decreased when the bacterial infection was eradicated. Wu et al, reported the existence of different pathways of gastric carcinogenesis in different histological subtypes of gastric cancer and its progression, in which *H. pylori *infection can play an important role [18]. Hibi et al, concluded that persistent *H. pylori *infection caused gastritis, with degeneration and regeneration of the epithelium that increased cell proliferation and the accumulation of p53 [19]. This in turn increased instability of the gene, as reflected by the development of carcinoma, using immunohistochemical methods to investigate p53 expression, and concluded that expression is associated with histopathological phenotypes, and that genetic alterations may not be affected by *H. pylori *infection [20]. Chang et al, noted the possibility that *H. pylori *infection and mutation of the tumor suppressor gene p53 may be significantly related with the process of gastric carcinogenesis in well differentiated and moderately well differentiated carcinomas [21]. However, Hongyo et al, claimed that *H. pylori *infection was more common in patients without any mutation in p53 [22].

The development of an enzyme-linked immunosorbent assay (ELISA) for mutant p53 protein makes it possible to determine most mutant p53 proteins in humans and other mammals [23]. This test has been used to determine mutant p53 protein in the serum of apparently healthy persons with *H. pylori *infection, detected as the presence of antibodies to specific IgG [24], beacuse most patients infected with *H. pylori *produce an easily identified systemic humoral immune responde, composed primarily of IgG. Circulating *H. pylori *antibodies persist at constant levels for years during infection.

Mutant p53 proteins have a half-life of approximately 24 h, whereas normal proteins have a half-life of about 20 min. It is this prolonged half-life which leads to the accumulation of detectable amounts of p53 protein [25].

Reactive oxygen species (ROS) are a group of highly reactive oxidative molecules implicated in the aging process, in several chronic inflammatory disorders, and in carcinogenic pathways in different epithelial districts [26]. An increase in cell ROS, be it due to overproduction and/or scavenging inability, may result in severe damage to various cell components, including membranes, mitochondria, and nuclear as well as mitochondrial DNA [27].

Ceruloplasmin (CP) is a 132 kd cuproprotein which, together with transferrin, provides the majority of anti-oxidant capacity in serum. Cp is a serum ferroxidase that contains greater than 95% of the copper found in plasma. This protein is a member of the multicopper oxidase family, an evolutionarily conserved group of proteins that utilize copper to couple substrate oxidation with the four-electron reduction of oxygen to water. Despite the need for copper in ceruloplasmin function, this protein plays no essential role in the transport or metabolism of this metal [28,29].

In this study, we sought to compare the relation between serum levels of mutant p53 protein and *H. pylori *infection in two populations of similar socioeconomic status, but with very different mortality rates for gastric cancer. A second objective was examine indirectly by measuring the serum concentration of the antioxidant ceruloplasmin in patients with evidence of *H. pylori *infection. Serum levels of ceruloplasmin usually vary inversely with serum nitrite levels [30-32].

## Materials and methods

### Type of study

This was a comparative, cross-sectional, case-control study of two populations with different rates of mortality from gastric cancer. This study has been ongoing since March 2002 to October 2005. Serum ceruloplasmin levels were also compared in patients with and without *H. pylori *infection, and in patients with and without mutant forms of p53. The investigators did not know whether the subject was positive or negative for *H. pylori *antibodies when they tested p53 status. For purposes of comparison, serum levels of *H. pylori *antibodies and p53 status were also determined in 71 patients with gastric cancer.

If *H. pylori *infection is related with cancer, the null hypothesis was that any variation or difference in seropositivity for the bacterium between the populations with high and low mortality rates due to gastric cancer is due to chance. The alternative hypothesis was that variations or differences in seropositivity between the two populations suggests that seropositivity for *H. pylori *infection is related with the rate of mortality from gastric cancer.

Ceruloplasmin, an organic antioxidant, is a marker for the presence of free radicals. We measured serum concentrations of ceruloplasmin and looked for correlations of these values with serum *H. pylori *antibody titers and p53 levels.

The objective of this study was to compare serum p53 values in a population characterized by a high rate of mortality due to gastric cancer and a high prevalence of *H. pylori *infection and a population with a low rate of mortality from this cause and a low prevalence of *H. pylori *seropositivity.

### Study populations

The population comprised inhabitants of two towns located 30 kilometers apart in the province of Cadiz (southern Spain), without prior treatment of *H. pylori *or who had recent eradication of *H. pylori *at least 8 weeks before were recruited. Although the socioeconomic level of the two towns is similar, Barbate is located on the Atlantic coast, whereas Ubrique is located in a mountainous inland area. We conducted a nutritional analysis and questionnaire survey for socioeconomic status in order to compare other risk factors that might influence *H. pylori *infection between groups. No significant differences in the nutritional factors or socioeconomic status, such as Hollingshead index, type of house, number of siblings, and crowding index, were found between the groups. Participants were permanent residents of these towns who were healthy and asymptomatic at the time of the study. Men and women aged 18 years and over were included.

The control group consisted in patients diagnosed with histologically confirmed gastric cancer, at the Departments of Internal Medicine, Medical Oncology and Surgery, of University Hospital Puerto Real from Cadiz. The median age of patients was 59 years (range: 33-85 years) and 57.5% of the patients in the series were male. Surgical specimens of 71 formalin fixed paraffin embedded gastric cancer with adjacent non-involved normal gastric mucosa were obtained from Pathology Department from our Hospital. Presence of tumor in the sections was confirmed by hematoxylin and eosin staining, and histologic typing of the tumors was performed according to both Lauren classification and WHO guidelines [33]. Specimens were examined by two independet experienced pathologists who also evaluated haematoxylin-eosin (H&E) and Giemsa stained slides for the presence of *H. pylori*.

Gastric cancers were classified according to the WHO classification as well differentiaded (n = 21), moderately differentiated (n = 25), poorly differentiated (n = 13), and signet ring cell carcinomas (n = 12). Evaluation of tumor stage was performed according to the criteria of the International Union Against Cancer (UICC) [34].

Subjects with a history of gastric surgery, dyspepsia, duodenal ulcer, gastric ulcer, malignancy, positive status for human immunodeficiency virus and/or hepatitis B, active gastrointestinal bleeding, or use of steroids or immunosuppressive drugs, H2 receptor blockers, antibiotics, bismuth compounds, or proton pump inhibitors or taking drugs interfering with free radical production (including vitamins C, A, and E, selenium and zinc) or similar nonprescription, were excluded. Were also excluded if they had had any disease for which reliable clinical information was not available, or if blood samples could not be obtained. Not more than two members of the same family were included.

#### Sampling procedure

We studied a total of 627 subjects: 308 from Barbate and 319 from Ubrique. Their ages ranged from 18 to 85 (median 55) years. For statistical analysis, were divided into 3 age groups; younger group (18-40 years; n = 101, median age = 29), middle-aged group (41-60 years, n = 197, median age = 53) and older group (≥ 61 years, n = 119, median age = 76). Sampling was random, and was stratified for these three age subgroups. Participants in this population study were visited at their home. All eligible subjects gave their informed consent for participation in this study and carried out according to the Good Clinical Practice guidelines and Helsinki Declaration.

#### Variables

As quantitative variables we recorded serum level of *H. pylori *IgG-specific antibody, expressed as IU/L [2,35], serum level of p53, expressed as ng/mL, and serum concentration of ceruloplasmin, expressed as mg/L [36]. As a nominal variable we recorded whether the subject was a resident of Barbate or Ubrique. As a dichotomous variable we used seropositivity/seronegativity for *H. pylori*, with a cut-off value of 51 IU/L.

A blood sample of 10 mL was obtained by venipuncture, and the serum was separated and stored at -80°C until analysis. Serum concentration of *H. pylori *IgG antibodies was measured with the Biolab Malakit (Wavre, Belgium) using an enzyme-linked immunosorbent assay (ELISA). In using this system, manufacturer's instructions were followed. *H. pylori *infection was defined as a positive ELISA result. The ELISA for serum p53 was from Oncogene Research (Calbiochem, Cambridge, MA, USA), that exclusively detected the mutant p53 protein, to eliminate a possibility of cross-reaction with other proteins, especially various inflammation-related products. This assay uses a mouse monoclonal antibody and a rabbit polyclonal antibody; the former reacts with an epitope located between amino acids 155 and 214 of the p53 protein, and binds exclusively to the epitope exposed on the mutant protein, but not on the wild-type protein. Therefore the assay is highly selective. All samples and standards were assayed in duplicate. *H. pylori *IgG and mutant p53 were quantified by extrapolating the average optic density for each set of duplicates on a standard curve obtained with known concentrations of purified *H. pylori *antibodies and mutant p53 respectively. For all analyses we used a Labinstruments SLT-400 ELISA spectrophotometer (Salzburg, Austria) with a 405 nm filter for *H. pylori *and a 450 nm filter for p53 [24]. Serum ceruloplasmin was measured by nephelometry with a Behring Nephelometer 100 analyzer (Behringwerke AG, Marburg, Germany).

#### Statistical analysis

All statistical computations were performed using SPSS software package (SPSS Version 10.0 for Windows, Inc, Chicago, IL) [37]. Descriptive statistics were calculated for each variable (means and confidence intervals). The statistical significance of the differences between groups were analyzed by Student's t-test or Mann-Whitney U-test. Significance of the difference between the seropositive and seronegative populations in towns with high and low mortality due to stomach cancer was found for serum concentration of p53 protein. The possible correlations between serum ceruloplasmin concentration, *H. pylori *IgG antibody level and p53 level. All tests of significance were 2-tailed, and a P value of 0.05 or less were considered statistically significant.

## Results

### Helicobacter *H. pylori *IgG antibody (Table 1)

In the coastal town of Barbate, 92 of the 308 subjects (29.87%) were positive for *H. pylori *IgG antibody, with a mean value of 242.5 IU/L (95% CI 232-386). Mean value in negative subjects (n = 216) was 19.4 IU/L (CI 16-24). In the inland town of Ubrique, 257 of the 319 subjects were positive (80.56%), with a mean value of 397.3 IU/L (95% CI 345-405 IU/L). The mean value in negative subjects (n = 62) was 16.6 IU/L (CI 12-22). The difference in the rate of seropositivity in the two populations was significant at p < 0.001.

**Table 1:** Serum concentration of anti-*H. pylori *IgG antibodies.

Population	N	Mean (IU/L)	CI 95%	p value
**BARBATE**	**308**	-------		
*H. pylori *(+)	92	242.5	232-386	<0.001
*H. pylori *(-)	216	19.4	16-24	
UBRIQUE	319	-------		
*H. pylori *(+)	257	397.3	345-405	<0.001
*H. pylori *(-)	62	16.6	12-22	
**GASTRIC CANCER**	71	-------		
*H. pylori *(+)	68	400	305-495	<0.001
*H. pylori *(-)	3	17.4	15-19	

CI, confidence interval

### Mutant p53 genotype (Table 2)

Of the 349 subjects who were seropositive for *H. pylori *IgG antibody, 286 (81.94%) had mutant p53, with a mean value of 0.973 ng/mL (95% CI 0.847-1.098). Of the 278 seronegative subjects, mutant p53 protein was detected in only 27 (9.71%), with a mean value of 0.239 ng/mL (95% CI 0.131-0.346). The frequency of quantifiable mutations was thus significantly higher in subjects who were seropositive for *H. pylori *IgG antibody than in seronegative subjects (p < 0.001). The mean serum value was significantly higher in patients with gastric cancer (1.973 ng/mL, 95%, CI 0.895-2.103) than in seropositive subjects (0.973 ng/mL) or seronegative subjects (0.239 ng/mL) (both p < 0.001).

**Table 2:** Serum concentration of mutant p53 protein and ceruloplasmin.

Population	N	Mutant p53 protein Mean (ng/mL)	CI 95%	Ceruloplasmin Mean (mg/L)	CI 95%
Overall *H. pylori *positive	349	-----	-----	477	435-519
HP (+) and p53 positive	286	0.973	0.847-1.098	486	439-532
Overall *H. pylori *negative	278	-----	-----	414	366-461
HP (-) and p53 positive	27	0.239	0.131-0.346	420	414-433
Gastric cancer	71	1.973	0.895-2.103	763	703-823

HP, Helicobacter pylori

### Serum ceruloplasmin (Table 2)

Of the 349 subjects who were seropositive for *H. pylori *IgG antibody, mean serum concentration of ceruloplasmin was 477 mg/L (95% CI 435-519). Of the 278 seronegative subjects, mean concentration was 414 mg/L (95% CI 366-461). Of the 286 subjects who were seropositive for *H. pylori *IgG antibody and who also had mutant p53, mean ceruloplasmin concentration was 486 mg/L (95% CI 439-532). This was significantly higher than in the 27 subjects who were seronegative for bacterial infection (420 mg/L, CI 414-433), with t = 2.23 (p < 0.05).

### Correlations between variables

We found no significant correlations between p53 and *H. pylori *antibody levels (R = 0.038) or between p53 and ceruloplasmin concentration (R = 0.139) in subjects who had anti-*H. pylori *antibodies.

### Patients with gastric cancer

Seropositive for *H. pylori *was detected in 68 of 71 patient (Table 1). Mean serum levels of mutant p53 in the 71 patients with stomach cancer were 1.973 ng/L (95%, 0.895-2.103). Mean serum concentration of ceruloplasmin in this group was 763 mg/L (95% CI 703-823). The mean level of mutant p53 protein in cancer patients was significantly higher than in healthy individuals who were seropositive for *H. pylori *infection (p < 0.001), but higher than in seronegative subjects (p < 0.01). (Table 2).

## Discussion

It is now accepted that *H. pylori *infection is a risk factor for stomach cancer. However, the mechanism of carcinogenesis associated with this bacterial infection in the stomach remains to be elucidated. The direct effects of *H. pylori *are certainly relevant to the induction of atrophic gastritis and cancer, and a number of virulence factors of *H. pylori *may have a role to regulate epithelial cell responses related to inflammation [38,39].

Our results show that among individuals with *H. pylori *infection, a higher than normal number also have elevated p53 protein. There appears to be a clear association between the presence of mutant p53 and seropositivity for *H. pylori*; however, prospective studies will be needed to demonstrate a causal relationship between the two phenomena. The mean serum level of mutant p53 protein that we found in persons with *H. pylori *infection was higher than the mean value in persons without infection, and was thus high enough to potentially facilitate the development of cancer. In those rare cases in which mutant p53 protein was found in seronegative individuals, the mean value was lower than in patients with stomach cancer.

It is a reliable laboratory procedure, since Shim et al, with the same laboratory procedure of mutant serum p53 measurement have got comparable but higher results in serum of cases with colorectal carcinoma [40]. The serum levels of mutant p53 are markers of tissular hyperexpression of this gene, as has been demostrated Suwa et al, in patients of pancreatic carcinoma [41]. On the other hand, Mukarami et al, shown the relationship between *H. pylori *infection and a direct sequence analysis of p53 gene mutation in a biopsy sample of human gastric mucosa, this finding appears to be involved in the pathway leading to dysplasia or carcinoma [42].

*H. pylori *survives in the host causes chronic inflammation by altering signaling pathways, downregulating inflammation, and dysregulating host immune responses. Carcinogenesis in the stomach is a multistage process; although p53 mutation is an important link in the chain, perhaps it is a promotion factor and other local initiating factors are needed for cancer to develop [15]. Our findings emphasize the importance of these additional carcinogenic factors, which are not directly related with p53 and were not investigated here. Although p53 mutation is a necessary factor, it is not in itself sufficient to trigger stomach cancer.

If a patient is found to be *H. pylori *positive it is important that the infection is eradicated because of the risk of associated pepti ulcers and gastric cancers. Prospective studies will disclose the fate of those subjects who are seropositive for *H. pylori *and who also develop significant levels of mutant p53. The results of such studies will make it easier to determine whether it is worthwhile to treat *H. pylori *infection in seropositive but asymptomatic persons; for now, it seems risky to declare, as do Konturek et al, [43], that prophylactic treatment is not indicated.

The presence of serum mutant p53 in itself provides no information on whether the mutation was the result of a genotoxic effect of the bacterium itself, or of a posttranscriptional alteration in p53 caused by bacterial toxins. Although the data from the present study do not shed light on this issue, the consequences for the p53 molecule are the same regardless of the mechanism involved.

Shiao et al, has been postulated that chronic atrophic gastritis, intestinal metaplasia and dysplasia are precancerous stages of stomach tumorigenesis and that mutation of p53 gene is an early event in stomach tumorigenesis [44]. Denaturation of the normal protein due to storage can be ruled out as the cause of the presence of mutant p53 in our subjects: all blood samples were processed in an identical manner regardless of *H. pylori *status.

*H. pylori *may exert a mutagenic effect on p53 through the production of free radicals in the cell. This hypothesis is supported by the concentrations of serum ceruloplasmin, an important organic antioxidant: mean concentration was higher in subjects who were seropositive for *H. pylori *and who also had mutant p53, than in subjects who were negative for both. Other studies have documented the presence of free radicals in the gastric mucosa of persons with *H. pylori *infection [45-47].

The contribution of p53 to the subsequent occurrence of gastric cancer was significant in *H. pylori*-seropositve subjects and non in *H. pylori*-seronegative subjects.

Oxidative damage is well documented in chronic gastric inflammatory diseases [48,49]. Recent published results showed that mucosal oxidative damage in H. pylori infection is associated with increased inflammatory cell infiltration, enhanced apoptosis, and cell proliferation, whereas it has been postulated that the progressive accumulation of oxidative DNA damage in certain genes, such as p53, may contribute to gastric carcinogenesis [26].

Such data suggest that apoptosis may be induced by both the transcriptional activation of a range of target genes and also by a range of other events that may presumably include signal transduction [50].

In summary, our findings suggest that *H. pylori *infection contributes to the development of gastric cancer by elevating the levels of mutant p53. However, although this may be a necessary promoter in the appearance of cancer, it is not in itself a risk factor in the absence of a previous triggering or initiating or mutagenic factor or factors and the other hand, the presence of anti-*H. pylori *antibodies in human sera remains one of the simplest methods of detecting *H. pylori *bacteria, and serological methods thus play an important role in the clinical practice.

## Abbreviations

ELISA: (enzyme-linked immunosorbent assay).

## Authors' Disclosures of Potential Conflicts of interests

The authors declare that they have no competing interests.

## Authors' contributions

JB, conceived of the study and participated in its design and coordination work. VG, AA and AL have made substantial contributions to patients sample collection and acquisition of data. GS, participated performed the statistical analysis. MD carrier out the ELISA studies. AS have made contribution to design, data analysis, interpretation of data, and drafting the manuscript. All authors read and approved the final manuscript.
